# Bibliographical Notices

**Published:** 1843-10-01

**Authors:** 


### Periscope;

OR,

### CIRCUMSPECTIVE REVIEW.

"Ore trahit quodcunque potest, atque addit acervo."

### Notices of some New Works.

PULMONARY CONSUMPTION, SUCCESSFULLY TREATED WITH NAPHTHA. By *John Hastings*, M.D.

DR. HASTINGS states, that the reason which induced him to deviate from that line of medical practice, which has so universally, and for so long a time, been in vogue, for that which he now brings forward, was the fatal termination of all cases, whatever was the treatment adopted, during an experience of upwards of twenty years.

From the greasy nature of tubercle in its crude state, Dr. Hastings concluded, that carbon entered largely into its formation, and that its composition had a striking resemblance to fatty matter. Among the changes in the earlier stages of pulmonary consumption, the disappearance of the fat is about the most remarkable; in consequence of this loss of fat, the author determined to employ those compound agents rich in carbon and hydrogen, which had not been previously used in medicine; "not with the idea that they would make up the deficiency which the system had sustained in the progress of the disease, but that such a change would by that means be introdúced into the constitution as would act upon the forces of the organism at the point of departure from health, whether that took place in the stomach, blood, or elsewhere;—that change tending to such an affinity in the elements within the body, that the carbon, hydrogen, oxygen and nitrogen, instead of assisting in the formation of products which threaten life, would tend to develope those materials only which are required for the perpetuation of health, and the prolongation of existence."

Accordingly, Dr. Hastings was led to employ naphtha as a remedy in pulmonary consumption. Many different compounds pass under this name, but the kind of naphtha termed *pyro-acetic spirit*, obtained by the destructive distillation of an acetate, generally of lead or lime, and in its outward form scarcely distinguishable from pyroxilic spirit, is the species which is considered to be the best suited for this purpose.

The following is the way in which Dr. Hastings employs the remedy.

"I administer naphtha three times a day, in doses of fifteen drops for an adult, mixed with a table spoonful of water, which is proportionably decreased according as the patient approaches youth. After the second or third day, I increase the dose by about one-fourth; regulating its increase or decrease, according to the absence or presence of nausea, sickness, or any other untoward symptom arising out of its use. As the disease advances, I increase the dose to forty and even fifty drops, and administer it four times a day, instead of three times.

"The successful use of naphtha, as an internal remedy, induced me to try its effects by inhalation, to which I was the more inclined from the results of the following experiments:—

"1st.—A little naphtha having been put into a bent tube, resembling the capital **U**, some expectorated matter was poured upon it, which had been determined with the microscope to be rich in globules of tubercle. Gentle heat was then applied, and the naphtha driven off, when the super-imposed secretion presented a mere shapeless mass of animal matter, the globules having entirely disappeared.

"2nd.—Some tuberculous secretion, highly charged with globules of tubercle, was placed under the field of the microscope, and a drop of naphtha added, when an immediate disappearance of the globules ensued, leaving behind a mass of the same character as on the former case. The frequent repetition of this experiment, invariably led to the same result.

"3rd.—Some tuberculous secretion of the lungs was put into a portion of the intestine of a child, and placed over a wide-mouthed bottle, which contained a small quantity of naphtha, between which and the intestine a clear space of three inches remained. A spirit lamp was then placed under the bottle, and a very gentle heat applied until slight ebullition took place, which was continued for an hour. The contents, when removed from the intestine and examined with the microscope, presented the same appearance as described in the previous experiments.

"Considerable benefit resulted from the inhalation of naphtha, in lessening the difficulty of breathing in the most advanced cases, in rendering muscular efforts less painful and fatiguing, and in a general alleviation of all those symptoms which distress the consumptive patient. The expectoration is not unfrequently rather increased immediately after the inhalation of naphtha, but the cough has changed for one of a milder character. Improvement was generally observed to follow that kind of inhalation which was performed with little exertion. It may be employed several times in the day, unless it produces nausea and sickness, when its use should be suspended; and on its being resumed, in such cases, it should be applied for a shorter period. When there is spitting of blood, its use is not admissible."

"Almost immediately after naphtha has been administered, an occasional rising of the medicine is perceptible in the mouth and throat, similar to that which occurs after a dose of castor oil. This is sometimes followed by nausea, and now and then vomiting supervenes. At other times it acts, but much more rarely, as an aperient. But when these effects occur, they usually subside in a day or two. It not unfrequently produces a glow in the region of the stomach, which extends over the chest and creates a sensation of cheerfulness and a greater freedom of breathing. It appears deserving a high rank among tonics; for in most of the cases in which it has been employed, a natural appetite was in a short time established. No remedial agent that I am acquainted with possesses such power over the colliquative perspirations of pulmonary consumption; as a few doses, in most instances, appeared sufficient to effect their removal. Another fact worthy of remark, is the absence of diarrhœa in all cases, which may be accounted for upon the supposition that tubercular deposit ceases to take place in the mucous track of the intestines. And even in those cases where diarrhœa, in the first instance, existed, it readily yielded to the naphtha treatment. The thirty-third case is a good example of this remark. Headache, particularly when the bowels are confined, is sometimes the effect of the naphtha treatment, and if aperients fail to give relief, a mustard poultice should be applied to the back of the neck, or a few leeches to the temples, or behind the ears. It will, however, be very seldom necessary to suspend the employment of the naphtha from this cause."

According to Dr. Hastings, from the very first moment he employed Naphtha in Pulmonary Consumption, up to the present time, it has been so successful in his hands, that he has no doubt it will be found, if used judiciously, to be little less than a specific in the earlier stages of the disease. And, from what he has more recently observed, he is most sanguine that, even in the latter stages of the disease, a restoration of health may generally be calculated upon.

"The progress of improvement in the physical signs, when sufficiently marked, invariably commenced with an amendment of the sounds arising from percussion. In no case did they appear to begin by those of auscultation; consequently I am induced to form the opinion that, as diagnostic signs, those derived from auscultation take precedence of those from percussion, or, in other words, that changes such as prolonged expiration, and very slight feeble and harsh respiration, may be detected by auscultation, when the sound elicited by percussion is not sufficiently appreciable to lead to any useful conclusion considered apart from auscultation."

Many cases are narrated in which the naphtha was employed.

If experience proves the correctness of these statements, Dr. Hastings will, no doubt, be considered a benefactor to the human race.

---

### On the Arrangement and Nomenclature of Mental Disorders. By *Henry Johnson*, M.D.

This Essay obtained the premium offered, in 1842, by the Society for the Improvement of the Condition of the Insane, for the best Treatise "On the Arrangement and Nomenclature of Mental Disorders."

The arrangement adopted by Dr. H. Johnson is a pathological one, and is as follows:—

Class.—Mental Disorders.

Order I.—*Congenital*:—Amentia.
- Idiotism.
- Imbecility.
- Cretinism.

Order II.—*Inflammatory*:—
- Phrenitis—*Acute.*
- Hypophrenitis—*Sub-acute.*
- Insania—*Chronic.*
    - (*a*) Moral Insanity—
        - Pathomania.
    - (*b*) Intellectual Insanity—
        - Mania.
        - Hypomania.
        - Dementia.

Order III.—*Non-Inflammatory*:—
- Delirium.
- Hypochondriasis.

Of the first Order we need say nothing.

*Hypophrenitis* is the delirium tremens of other authors. Dr. H. Johnson, however, considers this term as more expressive of its peculiar nature than the old; delirium tremens being, in his opinion, neither more nor less than phrenitis, but occurring from a peculiar debilitating cause, and in a constitution weakened by excess.

*Insanity.*—Dr. H. Johnson remarks, that proofs of inflammation have been discovered in protracted cases of insanity. "And, if the effects of inflammation be discovered in the brains of the Insane, surely Insanity, if not identical with, must be nearly allied to inflammation? I conclude, therefore, that the proximate cause of Insanity, is a chronic inflammation of the brain, and hence my reason for placing it in its present position in my arrangement."

Moral Insanity is the *Pathomania* of Dr. Prichard. It consists essentially, in a perversion of the usual habits and feelings, without delusion. The patient is wayward, eccentric, and extravagant in his conduct, but has no proper mental delusion or hallucination.

*Hypomania.*—This term Dr. H. Johnson proposes to substitute for monomania, as signifying a slighter form or lower grade of mania. "In this there is delusion, on one or more subjects, connected or unconnected. Sometimes the delusion is very persistent, sometimes it changes from day to day. The patient is, however, not violent, nor unmanageable. He has not the furious look of a maniac, but has more the appearance of a person in ordinary health."

*Dementia.*—This, in its last stage, fatuity, is characterised by the annihilation of the mental powers: it is to be distinguished from idiotism, by its not being congenital. It is usually the termination of mania. Dr. H. Johnson thinks that a new name, to be derived from the Greek language, might be advantageously substituted for the present one; he is, however, unwilling to suggest any unnecessary innovations of this kind.

*Delirium.*—This is classed amongst the non-inflammatory affections. The author looks upon delirium as always a symptomatic affection, depending upon some disease going on at the same time in the system. It appears to depend on an anormal condition of the circulation within the brain.

*Hypochondriasis.*—"This is chronic dyspepsia, attended with peculiar lowness of spirits, desponding habits of mind and undue attention to every uneasy bodily feeling. It is easily distinguishable from Insanity by the absence of any delusions, and by the prominence of the dyspeptic symptoms. It is said sometimes to lapse into Insanity, but this is not a frequent occurrence. The disease seems to commence with irritation of the organic or ganglial system of nerves, and the irritation is propagated by a species of reflex operation, first to the brain, producing the morbid state of the feelings, so characteristic of the disease, and from the centre of the nervous system, towards its extreme branches, in various parts of the body.

"I have no doubt that the tendency of the disease is hereditary; and, from what I have been able to observe, it appears to me, that the descendants of the ancient Cymri are peculiarly liable to it."

We see much, very much, to condemn, little, if anything, to approve of, in this proposed "arrangement and nomenclature of mental disorders."

---

THE IODATED WATERS OF HEILBRUNN IN BAVARIA, CONSIDERED WITH REFERENCE TO THEIR EFFICACY IN THE TREATMENT OF SCROFULOUS, CUTANEOUS, AND OTHER DISEASES. By Sir *Alex. M. Downie,* M.D. Physician to Her Britannic Majesty's Legation at Frankfort. Duodecimo, pp. 92, 1843.

This little volume consists chiefly of condensed extracts from a large work, (the 4th edition, of Dr. Wetzler,) on the same subject, by the learned German author who resides in the vicinity of a Spa which will probably take rank with Kissengen, Marienbad, or even Carlsbad, before many years.

The village of Heilbrunn is situated in a romantic spot in Upper Bavaria, between Flecken-Tölz, and Benedictbeuren, eight German miles from Munich. The *Quelle* of Heilbrunn rises between two eminences, and is said to be the oldest of all the Bavarian Spas. In 1659, it experienced a piece of good fortune, by contributing to the increase of the Elector's family, and its prolific virtues were speedily bruited about among the ladies, so as to establish its reputation. The neighbouring monks, however, became jealous of the Spa, and nearly ruined it, till, after nearly two centuries, (1805,) its renown was again revived by a German physician, (Graff,) who wrote a book on the waters. In 1825, Professor Vogel was employed to analyse the water, and he discovered the existence of iodine in the spring. This was afterwards corroborated by Wetzler. In compliment to the Queen of Bavaria, it now received the name of "ADELHEID QUELLE."

The following formula employed by Struve at Berlin, is supposed by Sir A. Downie, to be as nearly as possible, the analysis of the Spa in question.

(In the pint.)

| | |
|---|---|
| Iodate of Soda | 0,2000 |
| Bromate of Soda | 0,4090 |
| Muriate of Soda | 38,1540 |
| Carbonate of Soda | 6,8112 |
| Sulphate of Soda | 0,0072 |
| Carbonate of Potass | 0,2355 |
| Carbonate of Ammonia | 0,1203 |
| Carbonate of Lime | 0,6271 |
| Carbonate of Magnesia | 0,3974 |
| Carbonate of Strontian | 0,0517 |
| Carbonate of Baryta | 0,0221 |
| Carbonate of Iron | 0,0162 |
| Carbonate of Manganese | 0,0016 |
| Silica | 0,2562 |
| | 47,2906 |

The taste is decidedly saline, with an *après gout* of sulphur. The whole resembles a mixture of sea-water with that of Harrogate. It is perfectly clear, and suffers little by transportation. The spring does not throw up more than seven or eight hogsheads *per diem*, and therefore little can be spared for baths. The whole supply is nearly expended in bottling for exportation.

"The usual dose for children is from two to six ounces a day, and for adults as much as three quart bottles per diem have been ordered." 38.

"According to Geiger the Adelheid's Quelle may be prescribed with advantage in the following diseases.

"1st. Loss of Appetite and Indigestion.

"2nd. Chronic affections of the Liver and Spleen.

"3rd. In Ague and Jaundice.

"4th. In disease of the Kidneys from Calculous deposits.

"5th. In Chlorosis, Affections of the Womb and Sterility.

"6th. In Hypochondriasis and Melancholy.

"7th. In chronic Erysipelas, cutaneous diseases, as Psora, Porrigo, &c.

"8th. In gouty affections, stiff and enlarged joints, Palsy, &c." 46.

Cases, in illustration, are selected by the author from various German writers, and with these the little work concludes.

### Principles of Forensic Medicine. By *William A. Guy*, M.B. Cantab. Part I.

The contents of this part consist of Medical Evidence—Personal Identity—Age—Sex—Impotence—Rape—Pregnancy—Delivery—Fœticide—Infanticide—Legitimacy.

It appears to be plainly and sensibly written, containing much important information, condensed, without producing obscurity, into a small space. It is, of course, quite impossible to give any analysis of such a work, but we may extract the following history, drawn from the observations of Orfila, of the process of ossification as a means of determining the age of the dead.

"At 2 *months,* ossification of the os magnum, os cuneiforme, and os cuboide.

"At 4 *months,* ossification of the branches of the os hyoides.

"At 5 *months,* ossification of the lower apophyses of the os hyoides.

"At 6 *months,* an osseous point in the ensiform cartilage, and in the anterior arch of the atlas. Bony union of the body and alæ majores of the sphenoid bone.

"6 *months to* 1 *year.* Ossification of the cribriform and nasal plate of the ethmoid bone.

"1 *year.* An osseous point in the first bone of the coccyx, in the great tuberosity of the humerus, in the head of the femur and tibia, in the first cuneiform bone, in the coracoid process of the scapula, and two points in the odontoid process of the second cervical vertebra. There is bony union of the two portions of the posterior arch of the vertebræ, and of the several portions of the temporal and ethmoid bones.

"2 *years.* Ossification of the epiphyses of the metacarpal and metatarsal bones, an osseous point in the inferior extremity of the radius and fibula, in the transverse process of the seventh cervical vertebra, and in the base of the sphenoidal cells; and bony union of the two points of the odontoid process.

"2½ *years.* Patella and lesser tuberosity of the humerus ossified.

"3 *years.* Bony union of the body of the second vertebra with the odontoid process, and of the three portions of the fourth and fifth bones of the sacrum.

"3 *to* 4 *years.* Ossification of the great trochanter of the femur, the cuneiform bone of the carpus; and bony union of the styloid process of the temporal bone.

"4 *years.* Ossification of the second and third cuneiform bones.

"4 *to* 5 *years.* Ossification of the trapezius and lunar bones, formation of the ethmoid cells, and bony union of the body and processes of the second cervical vertebra.

"5 *years.* Ossification of the upper extremity of the fibula, of the epiphyses of the phalanges of the carpus, and of the epiphysis of the third phalanx of the great toe.

"6 *years.* Ossification of the lower extremity of the ulna, of the pisiform bone, and of the epiphyses of the first phalanges of the 2nd, 3rd, 4th, and 5th toes.

"7 *years.* Ossification of the internal condyle of the humerus, and of the first piece of the coccyx.

"7 *to* 8 *years.* Osseous point in the olecranon.

"8 *years.* Osseous point at the upper extremity of the radius.

"8 *to* 9 *years.* Ossification of the scaphoid bone of the carpus, and of the posterior epiphysis of the os calcis. Bony union of the two osseous points forming the head of the humerus.

"12 *years.* Osseous point on the inner edge of the lower articular surface of the humerus.

"13 *to* 14 *years.* Ossification of the lesser trochanter of the femur.

" 13 *to* 15 *years.* Three portions of the os innominatum firmly united by bone. Cartilages of larynx sometimes found partially ossified.

" 15 *years.* Osseous point in the inferior angle of the scapula. Bony union of the parts of the sacrum to each other, of the coracoid process to the body of the scapula, and of the two portions of the os calcis.

" 15 *to* 16 *years.* Osseous point in the summit of the acromion.

" 15 *to* 18 *years.* Osseous point in the sternal extremity of the clavicle, bony union of the sphenoidal plate to the body of the sphenoid bone, and formation of the spinous processes of the sacrum.

" 15 *to* 20 *years.* Ossification of the last bone of the coccyx.

" 16 *years.* Osseous point in the cotyloid cavity, in the head of the thigh bone, and in the head and tubercles of the ribs.

" 17 *to* 18 *years.* Ossification of the margin of the scapula. The epiphyses of the phalanges of the fingers and toes joined to the bodies of those bones.

" 18 *years.* Osseous points in the summit of the transverse and spinous processes. Bony union of the two trochanters and of the head of the femur to the body of the bone.

" 18 *to* 19 *years.* Bony union of the epiphyses of the metatarsal bones.

" 18 *to* 20 *years.* Bony union of the epiphyses of the metacarpal bones, of the lower extremity of the femur, and of both extremities of the humerus to the body of their respective bones.

" 18 *to* 25 *years.* Union of the body of the sphenoid to the occipital bone, of the three pieces of the tibia, and of the marginal epiphysis of the ilium.

" 21 *years.* Union of the lower extremity of the femur to the body of that bone.

" 20 to 25 *years.* Union of the first piece of the sternum to the rest of the bone, of the transverse and spinous processes of the vertebræ to their bodies, and of the tubercle of the rib to the body of the bone.

" 25 *years.* Formation of the laminar epiphyses of the iliac surface of the sacrum.

" 25 *to* 30 *years.* Complete union of the first to the second bone of the sacrum, and of the epiphisary discs of the vertebræ.

" 40 *to* 50 *years.* Union of the ensiform cartilage to the lower extremity of the sternum.

" 40 *to* 50 *or* 60 *years.* Union of the sacrum and coccyx.

The state of the osseous system will also furnish some clue to the age, especially during the latter periods of life. The internal cavities of the bones increase, from the absorption of the osseous matter, and the bones from the same cause become lighter. The bones of the head are solidly united, but, on account of the absorption of their diploe, become thin. The lower jaw becomes shallow, the alveolar processes are absorbed, and the angle again becomes obtuse, as in childhood. The spinal column is curved. The cartilages of the larynx and ribs are completely ossified. The osseous tissue generally is more dense, dry, and fragile, and abounds in earthy materials. In advanced age, moreover, the heart and arteries become more or less extensively ossified."

---

### The Vital Statistics of Sheffield. By *G. Calvert Holland*, Esq. M.D.

This is a very elaborate work on the statistics of the town of Sheffield, more especially as relating to the several branches of manufactures, the condition and habits of the workmen, and various points of local interest. We will extract some remarks on the fatal effects of "dry grinding."

*Fork-grinders.*—This occupation is perhaps more destructive to human life than any pursuit in the United Empire; it exceeds, however, only in a few degrees many other branches of grinding. Fork-grinding is always performed on a dry stone, and in this consists the peculiarly destructive character of the branch. In the room in which it is carried on there are generally from eight to ten persons at work, and the dust which is created, composed of the fine particles of stone and metal, rises in clouds and pervades the atmosphere to which they are confined.

"The dust which is thus every moment inhaled, gradually undermines the vigour of the constitution, and produces permanent disease of the lungs, accompanied by difficulty of breathing, cough, and a wasting of the animal frame, often at the early age of twenty-five. Such is the destructive tendency of the occupation, that grinders in other departments frequently refuse to work in the same room, and many sick clubs have an especial rule against the admission of dry grinders generally, as they would draw largely on the funds from frequent and long-continued sickness."

The mortality is tremendous. Of 61 persons, engaged in this occupation, 35 died under 30 years of age; 47 under 36 years. Only one attained the age of 48, and he had been fifteen years in the army before entering into this trade.

To form a proper idea of this mortality, it is necessary to compare it with the ratio of deaths at different periods in other classes.

"Thus in 1,000 deaths of persons above 20 years of age, the proportion between 20 and 29 years, in England and Wales, is annually 160. In Sheffield, 184; but among the fork-grinders, the proportion is the appalling number 475; so that between these two periods, three in this trade die to one in the kingdom generally.

"Between the ages of 30 and 39, a still greater disparity presents itself. In the Kingdom, 136 only in the 1,000 die annually between these two periods. In Sheffield, 164; but in the fork-grinding branch, 410; so that between 20 and 40 years of age, in this trade, 885 perish out of the 1,000; while in the kingdom at large, only 296. Another step in the analysis, and we perceive that between 40 and 49, in the kingdom, 126 die: in this town, 155; and in this branch, 115, which completes the 1,000. They are all killed off. For in carrying forward the inquiry we observe that between 50 and 59, in the kingdom, 127 die; and in Sheffield, 155: but among the fork-grinders, there is not a single individual left. After this period of life, there are remaining in the kingdom, of the 1,000, 441; and in the town, 339; but none in this branch of manufacture."

In 1841, ninety-seven adult workmen were employed in this trade. Of these ninety-seven, about thirty are at this moment suffering, in various degrees, from the disease peculiar to this occupation, viz. grinders' asthma.

"The disease is seated in the lungs and the air-passages, and the progress of it is accompanied with the gradual disorganization of these important organs. In its advanced stages, it admits neither of cure nor of any material alleviation. In the early stages, the only efficient remedy is the withdrawal from the influence of the exciting cause; but how is this to be effected by men who depend from day to day upon their labour, and whose industry from early life has been confined to one particular branch? Here, then, is the melancholy truth, that nearly one-third of this class of artisans, in addition to the poverty and wretchedness common to the whole, is in a state of actual disease—and disease which no art can cure. Fiction can add no colour or touches to a picture like this. Truth transcends the gaudy embellishments of imagination. The distempered fancy has here no room to exercise her powers."

### Some Account of the Epidemic of Scarlatina, which prevailed in Dublin, from 1834 to 1842 inclusive, with Observations. By *Henry Kennedy*, M.B.

For the last eight years scarlet fever has prevailed in Dublin to a great extent, and with symptoms of very considerable severity. Dr. Kennedy, who has had extensive opportunities of observing the epidemic, considers that it may be classed under the head of "scarlatina maligna;" not that every case put on the malignant form, but on account of the high proportion of deaths to the numbers attacked.

*Pathological Appearances.*—Dr. Kennedy divides the disease into two forms—first, the simply malignant; second, the complicated malignant.

In the first of these, the appearances on the surface of the body varied a good deal. In the greater number of cases, however, there were large dark petechiæ, or vibices, especially about the clavicles and groins. On cutting into these, little if anything was found to account for the mark so apparent on the surface; no extravasation was detected in any instance. In the head, chest, and abdomen, it may be stated generally that nothing but congestion, varying in degree in different cases, was found. The state of the throat in this form of disease was quite of secondary importance; during life it was often not complained of at all, after death some ulceration, of the upper part of the tonsils in particular, was found, unattended with any swelling.

The second form of the disease, to which Dr. Kennedy gives the name of "complicated malignant," owed its severity, in part, to the upper portion of the throat and its neighbourhood becoming engaged. In general the swelling of the neck occupied both sides; this swelling was often immense, encircling the neck like a cravat, and sometimes spreading down to the clavicles; in one case it engaged the pectoral muscles, rendering them as hard as a board. This tumefaction subsided considerably after death; on cutting into it in an early stage, effusion of serum, with a turgid vascular state of the lymphatic glands, was all that was found; at a later stage, pus of an unhealthy character was discovered, either infiltrated through the textures, or collected into a distinct abscess or abscesses. If the inflammation pursued the course of common abscess, the case usually did well: frequently, however, sloughing of the parts took place, proceeding with great rapidity, in some instances causing death by hæmorrhage from the coats of the large vessels becoming involved.

Sometimes the inflammation, after spreading down the neck, invaded the sterno-clavicular articulation, giving rise to abscess in the joint, or destruction of the cartilages.

Another form of swelling of the neck, to which the author directs attention, was caused by the effusion of nothing but lymph; this was rapid in its progress, at least to a certain point, for when the swelling had reached its height, it seemed at once to become stationary, in fact it rarely went on to form abscess. The hardness in these cases was excessive.

With regard to the condition of the internal fauces, two affections more particularly came under Dr. Kennedy's notice, viz. diphtherite, and œdema of the glottis. Both of these were accompanied by ulcerations, varying in extent and number—thus there was very constantly one in the upper part of either tonsil, with irregular edges, and of considerable depth. Ulcers were also found about the chordæ vocales.

*Sequelæ.*—In some cases, when the patient was to a certain degree convalescent, it was found that the child's neck had become crooked, and that when any attempt was made to bring the head straight, it caused severe pain referred to the upper part of the neck, and commonly to one side. This may have arisen, in some instances, from the inflammation of the throat spreading to the neighbouring textures, so as to engage those of the spine, and sometimes in this way cause caries: in other cases it occurred where there had been swellings of the neck, with the effusion of lymph, this effusion taking place about the small muscles of the nape of the neck, and sometimes in the upper portion of the trapezius; it was attended with some pain on pressure, and was very difficult to remove. In a third set of cases it arose from a spastic state of the muscles on one side of the neck, more particularly engaging the sterno-mastoid, and appeared to be caused by the patient lying in one position in bed for a considerable period.

Another sequela of the disease consisted in the inflammation spreading into the Eustachian tube, and so reaching the internal ear, causing abscess and exfoliation of the small bones. At other times, an abscess formed in the parotid region, and burst into the ear.

Purulent effusion into the joints was another and most formidable sequela of this epidemic. Sometimes only one of the large joints was attacked, at other times three or four were found to be full of pus. The synovial membrane was commonly healthy, though at times it was found intensely injected and coated with lymph. The same remark may be made of ulceration of the cartilages. In some very severe cases, the epiphyses separated from the shafts of the long bones. The internal organs were not affected in these cases.

The milder forms of the disease were frequently complicated by internal lesions. Of these the attacks of the chest were the most frequent and most serious. Dropsical effusions were also often present. In these cases, as a general rule, the kidneys were quite healthy, whether the urine was or was not albuminous. In other cases again, great congestion of these organs existed, sometimes to so marked an extent, that it appeared to have enlarged the kidney. In three instances, one of the stages of Bright's disease was very well marked; the kidneys were a good deal larger than natural, and their texture soft; and when the fibrous coat was taken off, the surface was distinctly mottled as if from the deposition of lymph.

*Symptoms.*—The invasion of the disease was generally sudden, the symptoms being commonly referred to the stomach. The stage of collapse rapidly followed, marked by its usual symptoms; there was seldom any distinct rigor, it was more of a creeping or shuddering, and often existed with a warmer state of the surface. The average duration of this stage of collapse was from two to five hours, when reaction set in, and the more prominent symptoms of the disease made their appearance. Sore-throat was constantly met with, varying from simple redness to the most extensive sloughing. Chronic enlargement of the tonsils, and an unnatural redness of the throat, remained for an indefinite time in some cases. The tongue very frequently did not present the usual appearance said to be so characteristic of scarlatina. The pulse was usually much quickened; during the stage of collapse, it was weak and indistinct; but in from eight to twelve hours it had again developed itself, and was to be found ranging close to 120. From this period it steadily declined in strength, till about the fourth day, when (supposing the patient did well) it again began to improve in tone and vigour, and its frequency became lessened. In those instances where the disease was more like a plague than scarlatina, that is where there was little if any reaction, it at times happened that no pulse could be felt at the wrist for several hours before death. The eruption varied considerably in its intensity; in its most severe form, it was in one continuous sheet over the whole surface. In other cases, it presented itself in great patches of a very bright colour, as large as one's hand would cover, but which were entirely separate one from the other. In both these cases spots of purpura occasionally appeared subsequent to the general eruption. If the eruption appeared in the following manner, the disease was sure to prove of a very severe form. "A case would occur in which the eruption of the common character had come fully out; it might or might not be favourable, but on the following day an additional crop would make its appearance; this was usually of a brighter colour, and much better defined than the first: and what was curious about it was, that this last eruption might disappear again, leaving the skin only covered with the first."

In many instances nothing but large dark petechiæ were visible about the clavicles and inguinal regions, often running down the inner side of the lower limbs. Sometimes there was merely lividity of the hands, feet, and depending portions of the trunk. In many cases, where there was very little eruption, the skin of the hands, particularly where the nails join it, assumed a very peculiar red and shining aspect, without any swelling. These were all very severe cases. As a general rule, the fever presented either the irritative or the typhoid form; very rarely indeed was it of the sthenic inflammatory type.

*Treatment.*—The line of treatment on which Dr. Kennedy placed most reliance, and which he used in the majority of instances, was as follows:—stimulant emetics—warm baths—tepid or cold sponging—cold affusion to head—internal stimulants—diluents—free application of nitrate of silver to the throat, followed by soothing treatment—occasionally the internal administration of opium. "Such, modified by the peculiarities of each individual case, was the plan of treatment adopted, and the one which, under analogous circumstances again, I would certainly have recourse to."

Numerous cases are detailed; and the volume closes with an appendix, containing an account of an epidemic of scarlatina which prevailed in the County of Armagh during the year 1842, communicated by Dr. Lynn, of the Market-hill Dispensary.

---

### Contributions to the Materia Medica. By *Fr. Simon.*

### I. Chemical Cautions in the writing of Prescriptions.

It is a prerogative of modern times, for which, according to some, we are indebted to homœopathy, that the writing of prescriptions is no longer conducted in that complex and confused manner, where for every symptom a remedy stood on paper, and where possibly one, two, or more remedies became ineffective by reason of mutual decomposition, or else exercised an entirely different effect from that intended; but now medicines are ordered in a form as little complex as possible, or even in a simple form altogether. Still, notwithstanding this essential improvement in the writing of prescriptions, it sometimes occurs, that the physician, deficient altogether in a knowledge of chemistry, unfortunately selects the few remedies he employs in such a manner, that they chemically destroy each other, and act either not at all, or in a different manner from what he expected.

Independently of the inconvenience hence resulting both to the patient and the cure in general, but which may be soon discovered and again rectified by a prudent physician well versed in the practice of such cases, we must now consider another inconvenience of a far more serious import. The recipe is in a certain way the only written document, which the physician gives to the apothecary and to the public at large with respect to his conduct and mode of treatment. The apothecary soon sees the faults committed against the laws of chemical affinity, and the public must observe them, when the medicine obtained, whether mixture, solution, &c. exhibits entirely different properties from what the physician assigned it. Now, though the apothecary is sometimes so complaisant as to take the blame to himself, this however does not always happen, and the physician necessarily loses in character.

The study of chemistry, considered from this point of view, is urgently necessary to the physician for daily use; but it embraces a field far too extensive to be acquired with anything like facility. Hence it occurred to us, that some general rules may prove not unacceptable, which may serve as a clue to enable practitioners to shun those gross chemical blunders in the writing of prescriptions.

### 1. SUBSTANCES MUST NOT BE PRESCRIBED IN SOLUTION WHICH ARE INSOLUBLE.

#### A. *The Aqueous Solution.*

As the aqueous solution is the most usual, the most frequent errors are committed in this instance. It is known that water dissolves the so-called soluble salts, extracts, gum, sugar and soaps, not resins, oils, fats, balsams, camphor, nor sulphur, phosphorus, iodine, calomel, &c.

With respect to the salts, or saline combinations, it is necessary to exhibit some which are insoluble or nearly so, though they are still prescribed in the form of solution; we must, however, impart to them solubility by means of other salts which are soluble; in cases of this kind errors are not infrequently committed.

The following substances are insoluble, or nearly so:—

Magnesiæ carbonas and m. usta—ferri prussias, et f. phosphas.—pulvis antim. compos.—antimonii sulphuretum auratum,—antim. sulphuretum rubrum,—zinci carbonas, zinci cyanidum, and some other metallic cyanides—to these, also, may be added the pure vegetable alkalies, quinine, cinchonine, salicine, and the sulphate of quinine, so often prescribed in solution; which is dissolved in pure water in extremely small quantity, but readily in water slightly acidified with sulphuric acid.

Of the combinations of iodine and bromine, those of iron are readily soluble—the same may be said of the iodide of zinc—iodide of gold is insoluble—the perbromide and protobromide of mercury are very difficult of solution—the periodide and iodide of mercury are insoluble.

With respect to the solution of extracts, it should be observed, that this is perfect in but few cases; partly because the extracts become changed, and a part is rendered insoluble by its combination with oxygen, partly because they contain resinous combinations, especially if they have been prepared by means of alcohol; in these cases the solution is turbid, being partly of a brownish, and partly of a greenish colour.

#### B. *The Alcoholic Solution.*

Alcohol chiefly dissolves resins, ethereal oils, balsams, soaps, camphor, and iodine. Of the salts, alcohol dissolves more especially the combinations of chlorine soluble in water, or bichloride of mercury, the chloride of gold, chloride of iron, &c.—the acetates and lactates, the carbonates, sulphates, and phosphates are not at all soluble in alcohol. Alcohol dissolves but a very small quantity of sulphur and phosphorus.

The alcoholic solution of resins, ethereal oils, balsams, camphor, and iodine are thrown down on the addition of water. The salts not soluble in alcohol, gums, also, several extracts, are thrown down from their aqueous solution, by adding alcohol.

#### C. *The Ethereal Solution.*

Sulphuric ether dissolves fat, ethereal oils, camphor, phosphorus, and some combinations of chlorine, as chloride of iron, chloride of gold, and chloride of mercury.

D. *The Oily Solution.*

The ethereal oils combine with fluid and solid fats; they dissolve camphor, sulphur and phosphorus, the Dippel oil in great quantity; they are soluble in alcohol. The fatty oils mix with the ethereal oils; they also dissolve camphor, sulphur, and phosphorus; in æther they are readily soluble, in alcohol but slightly so.

When a substance is given in solution, the most indifferent solvent must be employed, lest, when a full dose is to be administered, the action of the solvent should predominate.

2. NO SUBSTANCES SHOULD BE PRESCRIBED TOGETHER, WHICH MUTUALLY DECOMPOSE EACH OTHER.

This is a principle which may easily be defended, but it is one which must find a stumbling-block raised by usage. But why do we not endeavour to remove this block by the aid of science, a block, too, not set up by justice, but by mere custom? Were there no re-action in science, we should make no advance, and use and authority, however badly grounded, would become laws. We do not mean to say any thing here against the black and red wash, nor against Goulard's mixture, and several other preparations of that kind. These are useful preparations in the cases where they are indicated. We here intend to guard our readers against those unchemical formulæ where the intention, as well as the character, of the practitioner simultaneously suffer.

The following general rules may probably serve many a practitioner as a clue, wherein it is to be remarked, that in reference to the medicine which is to be prescribed, and that which is to be avoided, a reciprocal relation always naturally exists.

When salts of baryta, of lead and of lime are ordered, sulphates and free sulphuric acid are to be avoided, as the sulphuric acid yields white precipitates, difficult of solution with salts of baryta, of lead and of lime.

When salts of silver and salts of lead are prescribed, the hydrochlorates and free hydrochloric acid are to be avoided as the chloride of silver is insoluble, and the chloride of lead is difficult of solution.

When metallic salts are prescribed, care must be taken to avoid:—

*a.* Combinations of sulphur, especially those that are soluble, as sulphuret of potassium, or of ammonium, as the insoluble black or yellow, or reddish-coloured metallic sulphurets are precipitated. Even when both the metallic salt as also the sulphur-combination are insoluble, their combination should be avoided. Insoluble sulphur-combinations with such as are soluble, may be prescribed together.

*b.* Soluble carbonates, more especially carbonate of soda, carbonate of potash, as they form insoluble metallic carbonates; this is more especially to be remarked in the salts of iron, lead, copper, zinc, antimony and mercury.

*c.* The free alkalies and free earths, more especially the aqua ammoniæ causticæ, and the aqua calcis, are to be reckoned here: on mixing the metallic salts with these substances, the metallic oxides are generally thrown down in the state of hydrate. It is necessary to keep this in view when ordering substances for external use; as in ordering corrosive sublimate, calomel, acetate of lead, &c. &c.

*d.* Extracts, and especially those containing yellow colouring matter, (extr. of cascarilla, bark, rhatany, &c.)

All the metallic salts yield with the extracts, which, with few exceptions, contain a certain quantity of yellow colouring matter, insoluble, flocculent, dark-brown or black precipitates. These are particularly marked in the salts of mercury, gold, silver, lead, copper, and iron, so that, ordinarily, if the quantity of extract is sufficiently great, the entire metallic salt is decomposed. In the case of mercury the precipitate is dark-brown, in that of gold violet-brown, in silver brown, in lead a dirty brown, in copper a green brown, and in iron black. The narcotic extracts act of course in the same manner. In a combination of acetate of lead, chloride of mercury, or nitrate of silver, with opium or extract of aconite, we must always expect a portion of the metallic salt to enter into combination with the extractive matter and with the vegetable acids of the extract. Usage sanctions such formulæ, but the physician can never account to himself to which combination he is indebted for the favourable effect produced in the case under treatment; in a fomentation, in an eye-water, consisting of acetate of lead, and tincture of opium, it may be that the acetate of lead does not at all come into action, but the acetate of morphium, the meconate and the tannate of lead may be the real agents; the same thing holds good with respect to the composition of the collyrium, consisting of sulphate of zinc and acetate of lead, wherein the insoluble sulphate of lead is formed, and an acetate of zinc continues in solution. If the practitioner attain his end with this, so much the better. Only if he proceeds rationally, he should know, which substances produced the effect.

*e.* Soaps. Soaps are decomposed by the metallic substances; combinations of the metallic oxides with the fatty acids are separated: this is more especially to be kept in view in the salts of iron, lead, and copper. To order insoluble metallic combinations with soaps, is not at all warranted.

*f.* Vegetable mucus. It is the salts of lead and iron, more especially, which combine with the mucus, forming insoluble gelatinous masses.

In ordering free or carbonated alkalies or earths, the free acids must be avoided, unless in the one case of effervescing mixtures. Saturations should contain a slight excess of free acid rather than of carbonated alkali. Certain extracts, and those especially rich in salts, but above all extr. aloes cum acid. sulphur. in like manner, decompose the carbonated alkalies, or the ammoniacal combinations formed sometimes by their being kept for a long time in considerable quantity, are broken up, and ammonia is evolved. Hence, in prescribing pill-masses, in which carbonated alkalies and vegetable extracts, as the extract. taraxaci. card. bened. absinth. or the extracts of narcotic plants are contained, great caution is required. The pills in which this action of the constituents on each other, and the development of a gas takes place, become after a time larger and larger, and may swell from the size of a pea to that of a small hazel-nut.

In ordering *sulphur combinations*, more especially sulphuretted alkalies (sulphuretum potassæ) free acids or acid salts must be avoided. But it must be considered that in a neutral fluid an acid may be generated through fermentation, and particularly in substances containing sugar, as for instance in the syrup of white poppies. This circumstance merits particular attention when ordering the kermes with sugar as a linctus. If an acid is generated here in consequence of fermentation, the golden sulphuret is decomposed, sulphuretted hydrogen is formed, and an antimonial oxide, which acts as an emetic.

It has been already mentioned that in ordering *sulphur combinations* metallic salts in the reguline metals must be avoided. In this respect it is only to be remarked in the case of pills, that we must avoid directing, that the pills in which sulphur combinations exist be covered with gold or silver leaf, as in a little time the metallic covering becomes black.

With respect to the ordering of *metallic salts*, very different in reference both to their action and to their chemical habitudes, as for instance, of the chloride of gold, nitrate of silver, acetate or sulphate of copper, acetate of lead, chloride of mercury, &c. cautions have been given in the foregoing observations. As these substances are frequently ordered in the form of pills, but when decompositions are to be avoided extracts must be shunned, conformably to the experiments which I have formerly instituted on this subject, I propose as a pill-mass a mixture of equal parts of the pulvis althææ and of sugar, with the necessary proportion of water. When there is no reason to apprehend that the metallic salts will act prejudicially on the organs of deglutition, the form of powder seems most adviseable, and the saccharum lactis then appears to be the most appropriate constituent; however, in the case of corrosive muriate, chloride of gold, or nitrate of silver, the pilular form should be preferred. In solutions, however, these metallic salts, at least with respect to the eye, act still differently in reference to vegetable matters from what they do in the pilular form. An infusion of one ounce of chamomile flowers with 10 grains of acetate of lead or nitrate of silver forms two parts, one a precipitate, wherein is the metallic oxide as a metallic salt, and a fluid wherein there is little more than water. These purely empirical formulæ might probably be exchanged for others more judicious and rational, but it would be better, if physicians would make themselves acquainted with the remedies they order, (in the above case the combinations of the acetate of lead or nitrate of silver with the organic, extractive and other matters) as they know, for instance, in the unguentum ext. decub. antenr., that it is not the acetate of lead and yellow matter, but the tannate of lead that acts.

In ordering *the vegetable alkaloids* the xanthic acid and the vegetable substances containing yellow colouring matters must be avoided, as there are then formed insoluble combinations; hence it is, the substances containing yellow colouring matter are the most appropriate antidotes in cases of poisoning with the vegetable alkaloids. In the same case also iodine must be shunned; this too enters into insoluble combinations.

In ordering *soaps*, besides the metallic substances already mentioned, the earthy salts and the free acids and acid salts, as also the extr. aloes cum acido sulphur. must be avoided, by which latter remedies the fatty acids are separated; this is more particularly to be kept in view in ordering the sapo stibiatus from which not only the fatty acid is precipitated by the free acids, but also the sulphuret of antimony and hydrosulphuric acid gas is developed. A formula very prevalent in Berlin, but one by no means practically applicable, is the combination of the soaps with extr. al. c. acid. sulphur. corr. The fatty acid is constantly separated.

In like manner in ordering *the extracts* besides the *metallic salts*, the *free acids* must be avoided, by which a portion of the extractive matter is thrown down. Of the earthy salts alum is more especially to be remarked, which yields precipitates with the extracts, and more especially with the substances containing yellow colouring matter.

In ordering the *soluble earthy salts*, as *chloride of barium, chloride of calcium, sulphate of magnesia* and *alum, the carbonated alkalies* and phosphate of soda are to be avoided, by which free or carbonated or phosphated earths are thrown down. However it is to be remarked that the sulphate of magnesia is not thrown down by the bicarbonate of soda.

In ordering *the salts of potash*, more especially in the case of tartrate of potash, the free tartaric acid is to be avoided, by which bitartrate of potash is thrown down.

In ordering the simple tartrate of potash and also the double salts of tartaric acid, besides free tartaric acid other free acids also are to be avoided, more especially the mineral acids; the salts are decomposed in greater or less quantity, so that partly cream of tartar, and partly metallic combinations, are precipitated. But likewise *acid vegetable remedies*, as the pulp of tamarinds, &c., when they meet with the tart. tartaris., throw down cream of tartar. This accounts for the sediments which occur so frequently in these popular aperient mixtures.

On saturating common vinegar in saturations, or prepared vinegar, or fresh lemon juice, vegetable substances separate in the form of flocculi; hence it is that these saturations are turbid; but the saturation with distilled vinegar is clear.

The other saturations may be filtered, in which case however there is a loss of carbonic acid.

The combination of calomel with chlorine-combinations, more especially hydrochlorate of ammonia, is frequently productive of mischievous consequences, which is assigned to the formation of corrosive sublimate; this is possible through the medium of the vital process; however it has not been demonstrated by investigations that have been made on the subject outside the animal body. On the contrary, the combination of calomel with the so-called absorbent earths, as carbonate of magnesia or prepared chalk, seems to act prejudicially rather than beneficially, as by these carbonated earths carbonate of mercury is formed, which is obviously more energetic than calomel, and acts differently from it.

Some metallic salts are decomposed in consequence of the accession of the oxygen of the atmospheric air, and especially in the aqueous solution; this occurs chiefly with the salts of iron, as the chloride of iron, lactate of iron, and the ioduret of iron. To prescribe these substances in the aqueous solution is not advisable; their mixture with sugar-juice is to be recommended in preference, and equally judicious is their exhibition in powder with sugar of milk.

It is scarcely necessary to mention that deliquescent salts in powder should be ordered not in boxes, but in bottles. Such are more especially the salts of potash, the tart. tartarisat., tart. ammoniat., potass. carb. etc.

To mix ointments with aqueous or spirituous fluids is possible only to a certain quantity; if the quantity of the aqueous or spirituous solution is too great, it does not combine with the fat. Hence it is not possible to combine the linim. sapon. comp. with an ointment, as the former substance is a solution of soaps in spirit, whereas, on the contrary, the liniment. ammon. blends very well with ointments. If it be necessary to combine a great quantity of aqueous or spirituous substances with ointments, we must use the liquor ammoniæ causticæ as a connecting or binding medium, and partly saponify the fat.

In ordering phosphor. liniment, or ointments containing phosphorus, great caution is necessary that the solubility of the phosphorus in the fat be not overrated, otherwise particles of phosphorus continue undissolved, which may take fire on being rubbed in. One ounce of common fat dissolves from six to eight grains of phosphorus.

It is scarcely necessary to mention that when ordering eye-salves one cannot be too cautious with respect to the fatty acids of the salves ready prepared in the shops, which often baffle the best intention of the practitioner; for this reason an extemporaneous base of the salve should be selected, one prepared from almond-oil or wax. In ordering combinations of iodine, especially the iodide of potassium, the fatty acids must be avoided.

---

### A TREATISE ON MENTAL DERANGEMENT. By FRANCIS WILLIS, M.D. Second Edition.

Dr. Willis states that, the attention of the public having been of late much directed to the treatment of the insane, more particularly as regards restraint or non-restraint, he has been induced to re-consider the observations published by him many years ago on the "Cure of Mental Derangement;" the result is, that he is firmly convinced not only of the efficacy, but also of the necessity of restraint in the management of insane persons.

However agreeable, he says, the idea that a system of kind and watchful treatment is only required, he feels convinced that this will not *alone* avail. "The minds of the insane must be operated upon by control, either in fact or in idea. The greater number may, and certainly ought to be, rendered tractable without bodily restraint; but we must attribute the orderly behaviour we are able to produce, to a consciousness of being under restraint, which all patients feel when placed under the care of those, whom they soon find they must obey, by being obliged, from the first, to take their meals and their exercise, to go to bed, and to get up at the appointed hours; and, but for this fortunate circumstance, we should have great difficulty in keeping an otherwise ungovernable patient in a state of quiet subjection."

Dr. Willis is of opinion that it was to the early and prompt adoption of restraint that the great success of his grandfather is to be attributed; and to following the same mode of treatment he feels indebted for having been himself equally fortunate. Notwithstanding the outcry raised against restraint, and more particularly against "those beneficial auxiliaries, the waistcoat, the chair, and the belt and gloves," he does not shrink from upholding them, believing it is the abuse of these, and not the use, which ought to be condemned.

"The public, surely," the author says, "cannot know what these restraints are, against which so much prejudice is attempted to be raised. A strait-waistcoat is only a sort of jacket, made of any strong material, as stout drill, with very long arms and strings. When applied, the arms are folded over the chest, and tied behind. It takes full possession of a patient in the most lenient way possible, enables us to give him food and medicine, and prevents his doing mischief to himself, or to those who attend upon him; and, if judiciously applied, may be worn by the most delicate female without the slightest injury.—As to the chair, any strong chair with arms, so contrived that a patient cannot upset it, may be designated, 'the chair,' and is more particularly required when the patient is too restless to sit down, and would otherwise exhaust himself by standing. The belt and gloves, or the gloves alone, are a very useful as well as a very mild mode of restraint, and a good substitute for the waistcoat in those idiotic cases, in which the patient has a propensity to pick holes in his flesh, or pinch himself, or pull to pieces whatever he can put his hands upon." In short, he concludes that their use ought rather to be extolled as a mercy, than denounced as a degradation.

Dr. Willis conceives, also, that the late legislative enactments regarding the inspection of lunatic asylums, are calculated to increase considerably the numbers of the insane. Patients recently attacked, he says, are "grievously pained by the visitation, perhaps so seriously affected, as to have their recovery retarded or prevented altogether. What can be more heart-rending to a delicate mind than to be exhibited as a madman to these appointed inquisitors."

"The framers of these laws can never, I conceive, have contemplated the mischief they are likely to produce on this class of patients. A delicate female, even in a sound mind, could scarcely see four and five strange men enter her room without feeling some alarm; and can it be supposed that persons, whose nerves are already shaken, and who require to be kept free from excitement, can receive these strangers with impunity? The annoyance to those who are conscious of their state is more painful than I can describe. To speak even leniently of this measure, it is a cruel interference with the domestic affliction of private life."

With regard to the introduction of a system of religious instruction and discipline into lunatic asylums, the author is of opinion that it is a strange anomaly that it should be deemed useful to introduce into an asylum, for the daily attention of insane persons, a subject which is itself a very common cause of insanity.

"However desirable it may be, either for the sake of example or for the sake of preserving an orderly behaviour among the insane, that they should attend Divine worship, I am of opinion that the system of religious discipline should cease with that attendance. I think that all attempts at religious instruction are not only useless, but often injurious; and this opinion is fully corroborated by experiments which have been made on the subject".

Dr. Willis concludes by asserting that, as his practice has been principally confined to the affluent, his adoption of restraint cannot be ascribed to the want of means in providing adequate assistance in carrying out the most vigilant surveillance; but it is from a thorough knowledge and decided conviction of its peculiarly salutary effects that he most conscientiously recommends it, and strenuously urges the superiority of positive restraints over every kind of superintendence or watchfulness whatsoever.

"I am aware that I cannot expect by my observations to remove deep-rooted prejudices; but I may hope that reflective and judicious men, who are not themselves practical, and are anxious for information, will weigh the arguments here brought forward before they suffer themselves to be carried away with the stream, and yield their better judgment to theorists and philanthropists in opposition to practical experience."

It will be seen that the Doctor is a most decided opponent of the system of management now pursued by Dr. Conolly at Hanwell.

---

Practical Directions for the Preparation of Aerated Waters, and the various Compounds of Carbonic Acid Gas, &c. By *Robert Venables,* M.B.

Dr. Venables considers, that the chemical agencies of carbonic acid may be rendered extensively subservient to therapeutical pharmacy, from its capabilities of rendering some of our most powerful and active remedies soluble, and thus placing them in the circumstances most calculated to increase their energies, and insure their therapeutical agencies to the fullest extent and in the highest degree. Though some of the neutral compounds of carbonic acid, such as the carbonated alkaline earths, are wholly insoluble in water, yet water impregnated with carbonic acid will now prove a solvent. It is, however, principally to the preparation of proto-carbonate of iron, as a therapeutical agent, that our author's attention was directed.

"Carbonate of iron, though insoluble in water, yet was found singularly efficacious as a remedial agent. It is, however, a principle in pharmacology, that in general the power of remedies is not only exerted more quickly, but rendered much more active by solution. Therefore, to effect the solubility of the carbonate of iron forms an object of special moment. The salt, though insoluble in mere water, readily dissolves in water strongly impregnated with carbonic acid gas. This property consequently enables us to give steel under the much more grateful form of an *effervescing chalybeate.* This is effected by mixing together the equivalent proportions of sulphate of iron and carbonate of potass, or of soda, for decomposition and the generation of carbonate of iron. If carbonated water be drawn upon this mixture, decomposition will ensue, and the generated carbonate of iron be held in solution by the excess of carbonic acid. In this state it is fit for use.

"In the above process it is evident that with carbonate of iron we will have also sulphate of potass or of soda, as the case may be, owing to the mutual transfer of acids and bases. Notwithstanding that the quantity of alkaline salts, generally speaking, will be small, yet even so their presence is often a great inconvenience—sometimes even wholly inadmissible; yet under these circumstances they cannot be separated. It becomes therefore a question of some importance whether we can easily prepare an effervescing chalybeate, free from all other saline contamination. *By the aid of the carbonating apparatus, this is practicable, and both easily and quickly.*

"Carbonic acid condensed in water speedily attacks iron, forms it into carbonate and dissolves it. If then a small coil of iron wire, harpsichord wire for instance, be introduced into the pressure flask before its connexion with the apparatus; and the flask be now filled with distilled water, impregnated with carbonic acid under strong pressure, and allowed to remain so for some little time, a perfectly pure effervescing chalybeate draught will be obtained. Or if some iron filings or wire be placed in the water in the condenser, as the water becomes impregnated with the acid, the iron will be converted into carbonate, and held in solution, which will be ready for use the moment it is drawn off from the apparatus."

Dr. Venables states that he can, "from experience, assert that there is no preparation of iron so mild, so active, and so decidedly suitable as a chalybeate, or congenial to the animal economy, as the proto-carbonate of iron, held in solution by an excess of carbonic acid gas. If the digestion be impaired, provided there be no inflammatary action, the above preparation will be found to exert considerable influence. Should the blood be impoverished, and deficient in the red particles, this preparation will restore it to its healthy condition more effectually than any other. If we observe its action upon the pale and anæmious leucophlegmatic habit, we are often astonished at the rapid improvement, and the return of health and spirits."

The apparatus employed by Dr. Venables is Bakewell's patent portable aërating apparatus, a description of which is given, with instructions as regards its use for the preparation of aërated waters, lemonade, and the factitious compounds, &c. of carbonic acid. A long list is added, of diseases in which carbonic acid and its compounds may be administered with advantage; beginning with idiopathic fevers, and ending with incontinence of urine.

---

### Practical Remarks on Gout, Rheumatic Fever, and Chronic Rheumatism of the Joints. By *Robert Bently Todd*, M.D.

This work contains the substance of the Croonian Lectures delivered by the author during the present year.

The object which the author has had in view, he says, is to place in juxtaposition the leading facts in the Natural History of Gout and Rheumatism, in order to direct the attention of practitioners, in a more especial manner than has hitherto been done, to what he conceives to be their true pathology.

The work does not profess to give a complete history of these diseases; on the contrary, many details of symptoms, ætiology, and treatment, have been purposely omitted, as irrelevant to the argument, the design of which is, by contrasting the phenomena of gout and rheumatism with those of diseases confessedly caused by a morbid state of the fluids, to claim for them a similar origin.

We will proceed to notice a few points.

*On the Gouty Paroxysm, developing itself in depressed States of the System.*—Dr. Todd relates several cases tending to prove that, under certain circumstances, a feeble, or even exhausted condition of body, the very opposite indeed to that so often seen in acute gout, will favour the development and the recurrence of the paroxysm of this malady. It is not impossible that the prejudice against colchicum as tending to shorten the intervals between the paroxysms of gout, may be owing to the injudicious use of it in too large doses, which by their depressing effects may have favoured the more frequent recurrence of the fits.

One of the cases related is this; a gentleman of robust constitution, about thirty years of age, accustomed to active exercise, and who had lived pretty freely, had an attack of gout in the great toe; this yielded readily to treatment, and he took colchicum for a time. On his recovery he was directed to pursue a plan of diet, taking animal food in moderation, abstaining from malt liquor, and using wine in very small quantities. In the course of three or four months the gentleman had as many attacks of gout in the same toe. He had carried the plan of regimen too far; he had become a teetotaller, and frequently avoided taking meat; the consequence was, that he had become thin, pale, and enfeebled, and his muscles soft and flabby. He was recommended a good diet and tonic remedies; the gout left him, and he has not had an attack for two years.

Dr. Todd remarks, that he has noticed a peculiarity belonging to most of the cases of this kind that he has met with, namely, that the urine does not exhibit the abundant precipitate of the lithates which so often accompanies the gouty paroxysm. In some instances there was no precipitate at all; and in others it was very slight. The specific gravity of the urine was rather below than above the ordinary standard, indicating that no excessive quantity of either urea or lithic acid was held in solution.

"We learn, from such cases, two useful cautions. The first is, that in the treatment of the gouty paroxysm, we should be careful not to reduce the patient too low, lest a new paroxysm be induced. And secondly, that in the treatment of patients of the gouty diathesis for other diseases, we should be careful to avoid carrying the antiphlogistic regimen too far, for fear of exciting a fit of the gout."

*Heart-disease occurring in the Rheumatic Diathesis.*—Under the influence of cold, imperfect nutrition, or defective assimilation, the constitution is liable to be so modified, as to take on what may be called the *rheumatic diathesis*. This state Dr. Todd has observed to occur chiefly in children and persons under the age of thirty: seldom beyond that period, except where the diathesis existed in early life.

"It is characterized by the existence of a febrile state of the system, variously developed in different individuals, and indicated by quickness of circulation, occasional exacerbations evinced by heat of skin and perspirations more or less profuse; the perspirations having a sour odour. The urine is prone to the development of lithic deposits, more or less coloured. These symptoms, however, often escape the patient's observation (although readily detected by the attentive practitioner), and his chief complaint is of pains in the joints, not always occasioning swellings or enlargements, but often impeding motion; pains also in the muscles, or in the course of the nerves of the limbs; not stationary, but now affecting one limb or joint, and again another. The real nature of these pains is, I believe, often overlooked; and when they occur in children they are frequently regarded by nurses and others as due to the rapid growth of the child, and are popularly called 'growing pains.'"

This diathesis is also distinguished by marks of deranged nutrition. There is a cachectic appearance; the patient is thin, languid, keenly sensitive to vicissitudes of temperature.

In this state of constitution, the heart is apt to become affected; and the disturbance consequent upon this lesion may often be the first circumstance to excite the attention of the patient or his friends. The pericardium occasionally, but more frequently the endocardium and valves of the heart, are the parts affected. Dr. Todd has so frequently met with instances of diseased heart in young persons labouring under the rheumatic diathesis, but who have never had any actual paroxysm of rheumatic fever, that he cannot but regard this state of constitution as a fertile source of those cardiac diseases which are seen in early life. Hence he considers it most important for the practitioner to be on the watch for the signs of this state of constitution, as, by removing it, many young persons might be saved from the consequences of diseased heart.

*May the unhealthy Secretions of the Uterus afford Material for the Production*

*of Rheumatic Matter?*—When the blood is infected by certain foreign matters, symptoms are induced resembling those of a gouty or rheumatic kind. These morbid matters, we have good reason to believe, are primarily derived from a defect in that part of the digestive process which is performed in the stomach and duodenum. Several cases, however, have occurred to Dr. Todd which have impressed him strongly with the idea that the secretions of the uterus, if of an unhealthy character, and not duly thrown off, may be absorbed into the circulation, and contaminate the blood, producing symptoms of greater or less urgency, according to the nature and quantity of the morbid secretion which may have been absorbed. A case is related, in which there seems to have been an obvious connexion between the state of the womb and the rheumatic paroxysm, on the outbreak of which the uterine symptoms were relieved. The fever was of a low kind, and had the appearance of one produced by the poisoned state of the blood. The digestive organs had shown no sign of material derangement before the occurrence of the paroxysm; the part which seemed chiefly to suffer was the uterus.

The following is the proposition stated by Dr. Todd on this point. "In cases where the uterine secretions are imperfect, in quantity and quality, and are not duly thrown off from the organ, but are liable to become accumulated in it,—a condition which may sometimes occur in early life (*the rheumatic age*), or which may be deferred until the period when the menses are about to cease (*the gouty age*)—a diathesis may be met with among women strongly resembling the rheumatic or the gouty, and deriving its chief origin, although probably not its only one, from the abnormal secretion of the uterus."

The practical conclusion to be deduced from these remarks, would lead us to lay great stress on the adoption of all means calculated to prevent the retention of unhealthy secretions in the uterus, and to promote the healthy action of that organ; and the more so if the patient already show any indication of the rheumatic or gouty diathesis.

With regard to the treatment to be adopted in cases of gout and rheumatism, the author expresses very strong objections to the lowering system in gout; the following are his directions as regards the administration of colchicum.

"1. Colchicum should not be given in the asthenic form of gout.

"2. Colchicum should never be given at the onset of a paroxysm, nor until the bowels have been duly acted upon by mild purgatives.

"3. The first doses of the medicine should be very small; they may be gradually increased.

"4. Colchicum should be always administered at first uncombined with any other medicine, until the practitioner has satisfied himself that it is not likely to disagree with his patient. And indeed there is always a disadvantage in administering this medicine in combination with others; since it may become difficult, if not impossible, at times, to determine what effects should be ascribed to the colchicum, and what to the other ingredients.

"5. It should not be administered so as to excite nausea, vomiting, or purging. These effects should be regarded as indicative of the unfavourable operation of the medicine.

"6. Colchicum may be regarded as acting favourably, when, under its use, the urine is increased in quantity, a more abundant bile is discharged; when the fæces, though solid, are surrounded by mucus, and the skin secretes freely.

"7. The effects of colchicum should be carefully watched, as like digitalis and other medicines, it is apt to accumulate in the system.

"The use of this medicine seems chiefly applicable to the sthenic form of gout, which occurs in robust constitutions, and in the prime of life; but it is almost inadmissible in persons advanced in years, who have had several attacks, and in whom the malady would seem too deeply rooted, to be influenced by the temporary administration of this remedy."

In rheumatic fever, also, Dr. Todd has considered it his duty to protest against the too prevalent practice of large bleedings; other evacuants, he says, being as capable of modifying the fever, without the risk of doing ulterior mischief, and opium being more effective in relieving pain. The mistura guaiaci, as employed by Dr. Seymour, is a most useful medicine in this affection.

A work of considerable merit.

---

AUSTRIA: ITS LITERARY, SCIENTIFIC, AND MEDICAL INSTITUTIONS, &c. By *W. R. Wilde,* M.R.I.A.

The object of this work is stated by Mr. Wilde to be twofold: first, to afford information to the medical and general scientific reader in this country, on topics of considerable importance not hitherto inquired into, with respect to the state of medicine, the arrangement of hospitals and sanatory institutions, and the admirable system of education and *clinique* in a large portion of Europe. Secondly, this work may be found useful as a hand-book, in its particular province, to a country with which we are daily becoming more and more acquainted and connected.

We will notice a few points of interest as they arise.

*Division of the Medical Profession in Austria.*—At present, the medical profession in Austria is divided into the first class physicians and first class surgeons; the town and country surgeons, analogous to the general practitioners in Great Britain; those who practise specialities, as accoucheurs, oculists, dentists; the *pharmaceurs,* who are divided into the apothecaries and the doctors of chemistry; and, lastly, the veterinary surgeons—a class very superior to any other of a similar calling in Europe, and a large portion of whom are at the same time physicians and surgeons of the first grade. Each of these classes undergoes a certain fixed course of study. The education for the first class, physicians and surgeons, requires five years, and none are permitted to attend the lectures upon these subjects but those who have obtained, at the final examinations of their philosophical studies, a certificate of first-class in all the obligatory courses. They only are eligible to become doctors of medicine and surgery.

The second class surgeons, however, are in a far inferior position, being degraded, not only in letter but in spirit, to the mere barbers and dressers of wounds—a position from which they can never rise, no matter what their talents or abilities. Every *Wundarzt* (one of this class) is obliged by the law of the land to shave for a couple of *kreutzers,* exhibit the basin and striped pole, and keep open a barber's shop. And although many of these surgeons in the larger cities do not themselves manipulate upon the jaws and chins of the inhabitants, yet are they obliged to keep a servant or an apprentice to do so, as hair-dressers or any other class of the community, except the *Wundärzte,* are not permitted to perform this operation. These general practitioners are not allowed to sell medicine, but to them is committed the performance of all the minor chirurgical parts of the profession, such as usually falls to the lot of the apothecary with us; as for instance, bleeding, cupping, the application of leeches, and the dressing of simple wounds and fractures, &c. They cannot prescribe medicine, except a few simples, unless in cases of immediate danger, and when a physician or doctor of surgery is not at hand; they form, however, the principal practitioners in the distant country parts.

*Great General Hospital at Vienna.*—This most magnificent institution, the greatest either here or upon the Continent, contains within its walls 3,477 persons, and treats upwards of 30,500 patients annually. The object of this establishment is three-fold. First, to afford a comfortable asylum for those able to pay all, or a portion of their expenses; secondly, to provide in-door medical relief gratis for those unable to pay any portion of their cost; and, thirdly, to maintain a school of practical clinical medicine. The hospital is divided into three great departments—the medical and surgical hospital, the lying-in institution, and the lunatic asylum. There are 389 medical attendants to 3,025 beds, or one for every eight, independent of the different clerks and general servants not in immediate attendance upon the sick.

This hospital, however, with the exception of the lying-in department for females illegitimately with child, and its attendant institution for the foundlings born there, together with the female venereal wards, affords little or no *gratuitous relief.* The expences are defrayed by the patient himself or his friends, or in default of these, by the parish or district to which he belongs. In the event of a foreigner being obliged to seek medical relief in this or any other Austrian hospital, his ambassador is served with the bill of his expenses, which his country is obliged to discharge.

*State of Disease in Vienna.*—"Scrofulous diseases, particularly in the tubercular form, are more frequent in Austria that any other country I have visited. General surgery has but little to offer to the attention of the French or English student, except, perhaps, a lesson upon adhesive inflammation to the former. The want of machinery and manufactures, as well as the few heavy-wheeled carriages, and the sober quiet deportment of the inhabitants, compared with London, Paris, or Dublin, no doubt assists in lessening the number of accidents.

"Calculus and bladder affections are also comparatively less frequent than with us. I cannot praise the dressing and bandaging—it is neither neat nor scientific. The surgical divisions most deserving a visit, are those of Drs. Schuh and Moisisowitz, the most rising surgeons in Vienna. Rheumatism is a most frequent and dangerous affection in Vienna. Diseases of the skin are not very common in this part of Austria: there is a separate division set apart for their treatment, which is chiefly by baths and fumigations; but to those who have seen St. Louis, at Paris, it offers little attraction.

"The fever wards are usually crowded, especially in the cold season, and their mortality is very great; generally a third die; ulceration of the intestines, and scrofulous tubercular deposits in the abdomen, being the most usual pathological appearances. The visitor will have here a good opportunity of witnessing, on a large scale, the *Nerven fieber*, or true *typhus abdominalis* of the east of Europe. The treatment is more of the expectant than the anticipating plan, and consists, as indeed does the treatment of most diseases in this hospital, of some one line already marked out, and only deviating to meet symptoms as they present themselves, but never venturing upon new and unexplored ground. Stimulants are never used even in the advanced stage of this disease."

*Dr. Skoda's Clinique.*—This is purely a stethoscopic clinique for diseases of the chest, and is perhaps the best school for acquiring a knowledge of the diagnosis of such affections that the foreigner can visit. As an auscultator, Dr. Skoda possesses an unrivalled reputation, and certainly his diagnosis of heart and lung affections is astonishingly correct. It is entirely in this latter branch of knowledge that this clinique is so remarkable; for the treatment pursued there has in it nothing peculiar except that it is by no means good. It is purely antiphlogistic—consisting of blood-letting, leeching, and blistering, with the use of a few simples—such as tartarised antimony and the tinctura digitalis—administered in large doses. In pleuritic effusion he practises paracentesis much more frequently than any other physician, and does so even in acute cases; for he maintains that it must be performed early if at all, otherwise the lung having become collapsed and shrivelled up by the long-continued pressure of the fluid, will not again expand. He has also punctured the pericardium several times and with various success. In acute rheumatism he employs the constant application of iced water to the inflamed extremities, even where there is severe pericarditis present.

*Syphilis.*—The venereal affections in Austria appear generally to be of a milder nature than in Great Britain. This is probably owing to the sanatory police regulations, to the peculiarity of the climate, the temperate habits of the people, the non-use of mercury, and the non-existence of unqualified practitioners to attempt its treatment. True chancre is very rarely seen, and phagedenic and sloughing ulcers of the genitals are comparatively unknown. Among the secondary forms of syphilis, affections of the throat and papular eruptions are the most common, but syphilitic rheumatism and nocturnal pains are rare and less severe than in this country. Rupia, nodes, caries, the pustular form of eruption, the spreading syphilitic sore, diseases of the bones and testes, &c. and other severe secondary and tertiary forms are very seldom seen: indeed rupia appeared to be only known from English descriptions of it, and during six months Mr. Wilde did not see a single case of syphilitic iritis. Condylomata are very prevalent; these are supposed by the medical men there to constitute a primary affection, and to form in many instances the only symptom.

Inoculation with syphilitic virus, as practised by Ricord at Paris, is not permitted in Vienna. Infantile syphilis is exceedingly rare, and abortions from that disease are hardly known.

*Imperial Lying-in Hospital.*—This is probably the most curious institution in Europe. Pregnant women of every description can avail themselves of the advantages of this asylum; the poor and destitute are admitted gratis, and the rich by paying a certain stated sum. Every comfort is supplied—no visitor can intrude—*no law affect, and no authority reach its inmates—nay more, the very fact of their having been delivered there is inadmissible either as documentary or personal evidence in a court of justice.* The principle of secresy is imposed as one of the strictest duties on all those in any way engaged in the institution. Should a female desert her family, and take refuge here, the vigilance of the police or the inqniries of her friends may trace her to the door of the *Gebäranstalt,* but no further. Indeed in many instances, and in almost all the cases occurring among the highest class, a female may enter, accomplish her delivery, and depart from the hospital without her name being known, or even her face seen by the physician or any of the attendants. Persons are allowed to appear masked, veiled, or otherwise disguised; they may enter at any time previous to their delivery, and remain as long as they wish; they may carry their infants away with them or send them to the foundling hospital through the medical attendant. The names and address of persons admitted into this division are not required, but each female must write her name and residence upon a billet, which she seals, and on the back of which the physician inscribes the number of the room and bed she occupies. This ticket is then placed in a small locked-up cabinet beside her bed, and at her departure it is returned to her unopened; its object being, that in case of her death, the institution may inform her friends, or be able to produce this testimony of her decease on the demand of her relations or the police.

A considerable portion of the work is devoted to the consideration of the state of ophthalmic surgery in Vienna, but as this has been already noticed in this Journal*, we shall not again refer to it.

The volume is one of very considerable interest.

* No. 71, page 262.

